# Using AI to determine optimal cost-effective diagnostic pathways for chronic breathlessness

**DOI:** 10.1038/s41533-026-00548-9

**Published:** 2026-08-13

**Authors:** Max Olsson, Rafsan Ahmed, Joakim Ekstrand, Anders Blomberg, Tomas Jernberg, Mats Börjesson, Rachael A. Evans, Andrei Malinovschi, Jacob Sandberg, Magnus Sköld, Per Wollmer, Carl Johan Östgren, Gunnar Engström, Anders J. Björkelund, Magnus Ekström

**Affiliations:** 1 Lund University, Faculty of Medicine, Department of Clinical Sciences Lund, Respiratory Medicine, Allergology and Palliative Medicine, Lund, Sweden; 2 Cell Death, Lysosomes and Artificial Intelligence Group, Department of Experimental Medical Science, Faculty of Medicine, Lund, Sweden; 3 Faculty of Business, Kristianstad University, Kristianstad, Sweden; 4 Umeå University, Department of Public Health and Clinical Medicine, Umeå, Sweden; 5 Department of clinical sciences, Danderyd Hospital, Karolinska Institute, Stockholm, Sweden; 6 Dept of Molecular and Clinical Medicine, Inst Medicine, Sahlgrenska Academy, University of Gothenburg, Gothenburg, Sweden; 7 Center for lifestyle intervention, Region Västra Götaland, Dept Medicine, Geriatrics and Emergency Care, Sahlgrenska University Hospital, Gothenburg, Sweden; 8 Department of Respiratory Sciences, NIHR Leicester Biomedical Research Centre – Respiratory Theme, University of Leicester, Leicester, UK; 9 Dept of Medical Sciences, Clinical Physiology, Uppsala University, Uppsala, Sweden; 10 Department of Health, Blekinge Institute of Technology, Karlskrona, Sweden; 11 Karolinska Institutet, Department of Medicine Solna, Stockholm, Sweden; 12 Department of Respiratory Medicine and Allergy, Karolinska University Hospital, Stockholm, Sweden; 13 Department of Translational Medicine, Lund University, Malmö, Sweden; 14 Centre of Medical Image Science and Visualization (CMIV), Linköping University, Linköping, Sweden; 15 Department of Health, Medicine and Caring Sciences, Linköping University, Linkoping, Sweden; 16 Department of Clinical Sciences Malmö, Lund University, Lund, Sweden; 17 Lund University, Faculty of Science, Centre for Environmental and Climate Science (CEC), Lund, Sweden

**Keywords:** Computational biology and bioinformatics, Diseases, Health care, Mathematics and computing, Medical research

## Abstract

Breathlessness is a common symptom in clinical practice, yet evidence for cost-effective strategies to diagnose the underlying health conditions causing breathlessness remains limited. Using Swedish population data with individuals with moderate to severe breathlessness, we developed an artificial intelligence (AI) reinforcement learning model to identify optimal, low-cost diagnostic pathways for breathlessness tailored to subgroups based on sex and smoking exposure. Sixteen clinically relevant conditions were defined, along with their diagnostic tests and standard healthcare costs. The AI-based approach successfully produced efficient and low-cost diagnostic pathways for breathlessness with high diagnostic yield. The optimal sequences were overall similar between the subgroups. For all participant subgroups, the AI-derived pathways initially identified (or order of effectiveness in relation to costs) clinical evaluations of body mass index, anxiety and depression, physical activity levels, and spirometry. Subsequent steps included diffusing capacity measurements, chest computer tomography, and hemoglobin assessment. Overall, investigations of the lungs were prioritized ahead of investigations of the heart. This strategy has the potential to streamline the evaluation of breathlessness, reduce unnecessary testing, lead to an earlier diagnosis at lower cost, and support more targeted clinical management.

## Introduction

Breathlessness is a common symptom in the general population and is strongly associated with physical limitations, increased anxiety, depression, fatigue, social isolation, and impaired quality of life (QoL), and higher risk of premature death^1–6^. The symptom is a very common reason for hospitalisation and contact with primary care^7^. Multiple underlying health conditions that are common in the population may contribute to an individual´s breathlessness, including cardiorespiratory diseases, anxiety/stress, depression, and obesity^8–10^. Achieving a diagnosis is a key event to help access disease-specific treatments to improve symptoms, disease trajectory to reduce premature mortality, and disease-agnostic therapies such as pulmonary or cardiac rehabilitation. Purely symptom-based therapies for breathlessness remain limited^11,12^.

No study has evaluated the optimal diagnostic pathways for breathlessness^13^ while accurate, quick, and cost-effective ways to diagnose the underlying health conditions for breathlessness have recently been highlighted as a research priority^14^. Clinical evaluation of breathlessness is complex and often requires multiple tests in primary care followed by tests in specialised clinics^13,15^, which delays diagnosis and increases the economic costs for health care and the discomfort of the patients^16^. In primary care, where most patients are evaluated, resources are more limited than in specialised clinics which highlights the need for evaluation pathways that are evidence-based (in terms of effectiveness and costs) and targeted at primary care^16^. A recent review^15^ showed that the existing diagnostic pathways for breathlessness often suggest starting with basic tests in primary care clinics (such as spirometry) followed by more advanced testing such as chest computed tomography (CT). However, access to spirometry is often limited^17^, evidence is scarce on the economic efficiency of these strategies^15^, and the sample sizes of previous studies used for developing these strategies have been small^15^. Patients with breathlessness often have multiple underlying conditions contributing to breathlessness^8,18,19^, but with one exception^20^, all existing diagnostic pathways consider the work as “done” after identifying one underlying condition. This means that the reported accuracy percentage of identifying the correct conditions using many of the existing pathways can be misleading, but also potentially disadvantageous for patients with unknown and untreated underlying conditions. As developing accurate diagnostic pathways to identify multiple potentially co-existing underlying conditions is complex, novel artificial intelligence (AI) methods could be useful^13^.

The aim of this study was to develop a diagnostic pathway for chronic breathlessness identifying relevant underlying medical conditions efficiently and at low economic cost.

## Methods

### Study design and population

This was a cross-sectional analysis of the population-based multicentre Swedish CArdioPulmonary bioImage Study (SCAPIS) of 30,154 men and women^21^. Between the years 2013 and 2018, people aged 50 – 65 years living in or around the six study centres Lund/Malmö, Gothenburg, Linköping, Stockholm, Uppsala, and Umeå were randomly invited to participate in the SCAPIS study. Participants were invited to participate in the SCAPIS study regardless of health status.

Inclusion criteria for the present analyses were self-reported breathlessness defined as modified Medical Research Council (mMRC) breathlessness scale rating ≥2, corresponding to being breathless when walking on level ground, or worse^22^. Exclusion criteria to participate in the SCAPIS study were inability to understand written and spoken Swedish, and exclusion criteria for the present analysis were missing data on breathlessness (mMRC), or inability to walk for reasons other than breathlessness.

### Assessments and definitions

The SCAPIS data collection is detailed elsewhere^21^. At the study centres the following data were collected: survey data (mMRC breathlessness scale, questions about anxiety and depression, smoking history, physical activity, self-reported physician-diagnosed medical conditions), blood tests (Haemoglobin [Hb], N-terminal pro b-type natriuretic peptide[NT-proBNP]), physiological measurements (forced expiratory volume in one second [FEV_1_], forced vital capacity [FVC]^23^ diffusing capacity for carbon monoxide [DLCO], body mass index [BMI]), and computer tomography (CT) of the heart and lungs.

### Underlying conditions, diagnostic tests and costs

**Fig. 1:**
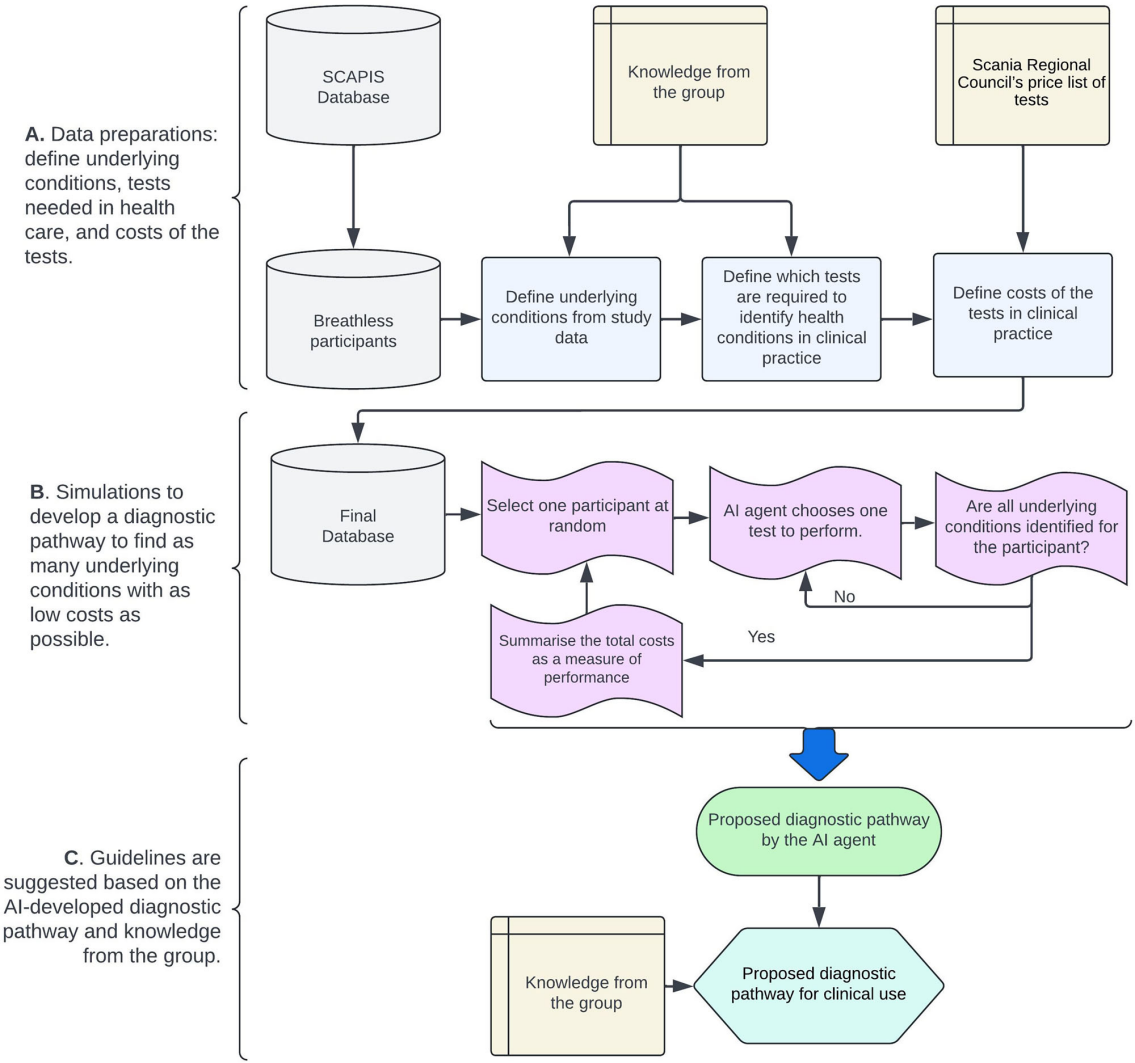
Study outline and analytic steps. **A**. Participants with breathlessness (modified Medical Research Council [mMRC] breathlessness scale rating ≥2, corresponding to having breathlessness when walking on level ground, or worse) were selected from the SCAPIS database. Data preparations include coding the underlying conditions and defining the tests which are required to identify the underling conditions in clinical practice with definition suggested by the group. The costs for the tests are then defined by Scania regional price list. **B**. In the final database, simulations are made to develop a diagnostic pathway to identify as many underlying conditions with as low costs as possible. First one participant is selected at random, then the agent chooses a test to perform. If all underlying conditions are identified, the agent receive the total costs of all used test as a measure of performance. If there remain unidentified underlying conditions, the agent will perform a new test. **C**. After one million iterations, a diagnostic pathway was developed. With knowledge from the group, guidelines are suggested for evaluating breathlessness in healthcare.

The process of defining underlying conditions, diagnostic test and costs is presented in Fig. 1. Underlying medical conditions known to contribute to breathlessness were identified based on subject matter knowledge of the authors and literature^8,9^. A spreadsheet was created by the first author comprising underlying conditions related to breathlessness identified in a narrative literature review^8^ and an epidemiologic study using the SCAPIS database^9^ and was independently assessed by authors (MB, GE, ME, MS, TJ, PW, JS, CJÖ, RE) from different expertise fields (clinical physiology, respiratory, cardiology, and family medicine) to add conditions that could contribute to breathlessness. In the spreadsheet, the authors were also asked to define test(s) that would be used in clinical practice to best identify each condition, to be able to calculate the costs of evaluating breathlessness in real health care. Standard costs of each test needed to identify the underlying conditions in clinical practice were derived from Scania Regional Council´s latest (2022) price list for tests^24^.

In the analysis, a primary care visit was considered the start point for patients seeking help for breathlessness and the costs of the tests that can be done at the primary care visit such as weight measurements and questions about anxiety, depression and physical activity were therefor counted as zero as these tests would normally not cost anything additional to the visit itself (such is the case in a Swedish context). Additional tests that are not included on standard in primary care are included in the costs (including spirometry, lung volumes and diffusing capacity, blood sample, ECG, echocardiography (UCG), NT-pro-BNP, CT chest, CT angiography and stress echocardiography). The input from the authors was then summarised by the first author and the underlying conditions present among the participants were coded based on the definitions in accordance with previous studies and are listed in Table 1. Participants with a missing value (mean among health conditions: 3%, range: 0 – 9%) on a health condition were assumed to not have that specific condition and the health condition were coded as absent.

**Table 1:** Underlying conditions associated to breathlessness, clinical tests to identify each condition, and their costs.

Condition	Test(s) used to detect the presence of the condition in the SCAPIS database	Test to identify the condition in clinical care (only used for cost estimation and not for definition/diagnosis in this study)	Cost of the test(s) needed to identify the condition in clinical care, SEK and Euro (year 2024)	
Obstructive lung disease (OLD)	Post-bronchodilator FEV_1_/FVC < LLN according to the GLI 2012 standard^28^	Spirometry	873 SEK	€76
Restrictive spirometry pattern	Post-bronchodilation FVC < LLN and FEV_1_/FVC > = LLN according to the GLI 2012 standard^28^.	Lung volumes	2209 SEK	€193
Asthma	Self-reported of physician diagnosis.	Spirometry	873 SEK	€76
Anaemia	Hemoglobin < 120 g/L for women and <135 for men^29^	Venous blood sample	289 SEK	€25
Obesity	BMI ≥ 30^29^	Weight	0 SEK	€0*
Underweight	BMI < 19^29^	Weight	0 SEK	€0*
Atrial fibrillation or other arrhythmia	Self-reported physician diagnosis	ECG	486 SEK	€42
Low physical activity	Self-reported that they are mostly sedentary during their free-time when asked about physical activity: “Sedentary free-time, (You spend most of your time with reading, TV, computers or other sedentary activities)”^9^	Question about physical activity	0 SEK	€0*
Heart failure	NT-pro-BNP value > 125 pg/mL (upper limit of normal [ULN])^30^ or self-reported physician-diagnosed.	Echocardiography, NT-pro-BNP	2648 SEK	€231
Pulmonary fibrosis (PF) or Fibrotic interstitial lung abnormality (FILA)	Pulmonary fibrosis = presence of honeycombing on lung CT; Fibrotic ILA = presence of honeycombing and/or reticular pattern with bronchiectasis on lung CT^33^.	CT, lung volumes, and diffusion capacity	5501 SEK	€481
Depression	Question about depression in SCAPIS; “yes” on the question “During the past twelve months, was there ever a time when you felt sad, blue, or depressed for two weeks or more in a row?” and “yes” on at least five of the following questions: “1. ‘Lost interest in most things like hobbies, work or activities that usually give you pleasure?’ 2. ‘Felt tired or low on energy?’ 3. ‘Gained or lost weight?’ 4. ‘Trouble falling asleep?’ 5. ‘Concentration problems?’ 6. ‘Thoughts about death?’ 7. ‘Bad self-esteem/feeling worthless?’^19^	Questionnaire	0 SEK	€0*
Anxiety	Assessed by the question “By stress we mean feeling tense, irritable, anxious or having sleeping difficulties as a result of conditions at work or at home. Did you experience this?” No stress was defined as answering: “never”, or “any stress period” and presence of stress was defined as answering “some stress periods during the last five years”, “constant stress during the last year”, or “constant stress during the last five years”.	Questionnaire	0 SEK	€0*
Lung emphysema	The presence of emphysema defined as at least mild emphysema (grade 1) in any location of the lungs^34^.	CT	3292 SEK	€288
Ischemic heart disease	Self-reported PCI or by-pass-operation and previous heart attack.	ECG and CT angiography or stress echocardiography	3892 SEK	€340
Reduced diffusion lung capacity	D_L_CO < LLN, calculated for sex and haemoglobin^35^	Lung volumes and diffusing capacity	2209 SEK	€193
Valvular heart disease	Self-reported physician diagnosis, or Aortic valve calcification found on CT.	CT	3292 SEK	€288

The costs are presented in Swedish Krona (SEK) or EURO (the exchange rate was determined as 1 EURO = 11,44 SEK at 2024-05-31). The following underlying conditions for breathlessness were defined based on previous studies: underweight/obese and anaemia^29^ on WHO standards, Chronic airflow limitation^28^, restrictive spirometry pattern^36^, pulmonary fibrosis (PF) or Fibrotic ILA (FILA)^33^, reduced diffusion lung capacity^35^, and pulmonary emphysemia^34^, anxiety, depression^37^, and heart failure^30^. Since the variables used in SCAPIS to define underlying conditions had the purpose of scientific research and might not be the same as would typically be used for the identification of underlying conditions in health care, these tests are separated and often different to be able to base the analysis on realistic economic costs of tests used in health care.

*BMI* body mass index, *CT* computer tomography, *DLCO* Diffusing capacity for carbon monoxide, *ECG* electrocardiogram, *FEV1* forced expiratory volume in 1 second, *FVC* Forced vital capacity, *GLI* global lung function initiative, *ILA* Interstitial lung abnormalities, *LLN* lower limit of normal, *NT-pro-BNP* N-terminal pro b-type natriuretic peptide, *PCI* Percutaneous coronary intervention, *SCAPIS* multicentre Swedish CArdioPulmonary bioImage Study, *WHO* World Health Organization.

*costs are included in a primary care visit and are there for not included in analysis.

### Analyses

Baseline participants’ characteristics were summarised using descriptive statistics. To be able to develop separate diagnostic pathways for clinically relevant patient groups, the participants were categorised into four groups by sex and smoking exposure of <20 versus ≥20 pack-years - a commonly used dose–response threshold for increased risk of morbidity^25^.

MO, RA, and AJB have a background in AI in medicine and gave input on the analysis and oversaw the training of the agent. To find an optimal evaluation strategy for identifying the underlying condition(s) related to breathlessness in terms of economic costs, we created a simulated environment in which a Reinforcement learning (RL) agent operates and a reward score is given the agent to improve itself (for details see Supplementary materials). The simulated environment refers to a software developed simulation in which the RL agent can act and get new information to learn a given task.

The analysis steps are shown in Fig. 1B. The AI agent was iterated for one million tests with the goal of identifying as many underlying conditions with as low costs as possible. First, a participant is chosen randomly from the dataset. Second, the agent is presented to which patient group the participant belongs to, and which tests have been performed so far. Third, the agent chooses a test to perform to identify an underlying condition. Fourth, the environment presents if all the participant’s underlying conditions are identified or if there remain underlying conditions to be identified. If all underlying conditions are not identified yet the agent goes back to the third step and continues with a new test. Fifth, if all the underlying conditions are identified for a participant, the environment presents the reward score which lets the agent know how well it performed. The agent then goes back to the first step and starts over with a new participant. With this approach, the developed diagnostic pathways are based on the prevalence of medical conditions among breathless patients as well as economic costs for tests in health care. The AI approach and how it was validated is detailed in Supplementary materials.

The diagnostic performance of each diagnostic pathways was evaluated as the percentage of participants for whom all their underlying conditions were identified after each step, for all participants in the whole dataset, by sex, and by smoking group.

### Consensus of clinical diagnostic pathway

Based on the diagnostic pathway developed by the AI agent, the multidisciplinary author group discussed and presented a diagnostic pathway to evaluate breathlessness proposed for clinical use. This proposed diagnostic pathway includes suggestions for physical examinations according to the International Primary Care Respiratory Group’s Desktop Helper^26^. Details about the procedure of reaching a consensus of the final diagnostic pathway is described in the Supplementary materials.

Diagnostic performance of this final diagnostic pathway was estimated based on the diagnostic % of the tests used in the AI developed pathway. Cost of the final diagnostic pathway was estimated by the costs of the tests stated in Table 1.

### Ethical considerations

SCAPIS was approved as a multicentre study by the ethics committee at Umeå University (Dnr 2010–228-31 M) and the present analysis was approved by the Swedish Ethical Review Authority (reference number: 2021-00288). All participants provided written informed consent.

## Results

### Participant characteristics

We included 1209 participants (69% women) with breathlessness (mMRC ≥ 2) in the analyses (**Supplementary** Figure S1). Participant characteristics and the prevalence of health conditions are presented in Table 2. Participants had a mean (standard deviation [SD]) age of 58.2 (4.4) years, BMI of 31.0 (6.0) kg/m^2^, with 224 (19%) being current smokers and 501 (42%) ex-smokers, and a mean smoking exposure of 13.8 (17.3) pack-years among all participants. The most common underlying conditions were anxiety (72%), obesity (54%), depression (38%) low level of physical activity (35%), asthma (20%), Obstructive lung disease (OLD) (18%), heart failure (18%), reduced Diffusing Capacity of the Lungs for Carbon Monoxide (DLCO) (17%), and valvular heart disease (12%). Among participants with ≥20 pack-years of smoking, there was a higher prevalence of underlying health conditions among men (mean: 3.94, SD: 1.76) and women (mean: 3.83 SD: 1.74) compared with men (mean: 3.11, SD: 1.64) and women(mean: 2.96, SD: 1.49) with <20 pack-years of smoking. Overall, men and women had similar prevalence of underlying condition, but asthma was more common in women compared to men, and men with ≥20 pack-years of smoking more often had low level of physical activity as an underlying condition (Table 2).

**Table 2:** Characteristics of 1209 people with clinically significant breathlessness from the Swedish general population.

	All participants	Men with < 20 pack-years of smoking	Men with ≥ 20 pack-years of smoking	Women with < 20 pack-years of smoking	Women with ≥ 20 pack-years of smoking
**N**	1209	238	141	608	222
**Age**	58.2 (4.4)	57.8 (4.4)	58.8 (4.1)	57.9 (4.5)	58.83(4.1)
**Women**	830 (69%)				
**Education level**					
No elementary school	24 (2%)	4 (2%)	2 (1%)	14 (2%)	4 (2%)
Elementary school	210 (18%)	42 (18%)	32 (23%)	89 (15%)	47 (21%)
Upper secondary school or professional education	618 (52%)	130 (55%)	87 (62%)	274 (46%)	127 (57%)
University degree	349 (29%)	60 (25%)	20 (14%)	225 (37%)	44 (20%)
**BMI (kg/m**^2^**)**	31.00 (6.0)	31.02 (5.6)	30.7 (5.5)	31.3(6.1)	30.3 (6.1)
**Current smoking status**					
Regular	169 (14%)	9 (4%)	57 (41%)	26 (4%)	77 (35%)
Occasional	55 (5%)	15 (6%)	8 (6%)	17 (3%)	15 (7%)
Former	501 (42%)	79 (34%)	74 (53%)	220 (37%)	128 (58%)
Never	467 (39%)	132 (56%)	0 (0%)	335 (56%)	0 (0%)
Pack years of smoking	13.8 (17.3)	4.2 (6.3)	39.3 (15.5)	4.1 (6.1)	34.4 (11.8)
**FEV**_**1**_ **(L)**	2.7 (0.7)	3.4 (0.7)	3.0 (0.8)	2.5 (0.5)	2.3 (0.5)
**FEV**_**1**_ **(%pred)**	97.3 (18.0)	97.05 (17.65)	88.31 (20.11)	101.2 (16.0)	92.52 (18.86)
**FVC (L)**	3.6 (0.7)	4.4(0.9)	4.3 (0.8)	3.2 (0.6)	3.2(0.6)
**FVC (%pred)**	101.9 (14.8)	99.82 (15.35)	97.69 (14.45)	103.46 (14.19)	102.51 (15.52)
**FEV**_**1**_**/FVC ratio**	0.76 (0.09)	0.76 (0.08)	0.71 (0.12)	0.78 (0.07)	0.72 (0.10)
**Underlying medical conditions**					
Number of underlying conditions, mean (standard deviation)	3.26 (1.65)	3.11 (1.64)	3.94 (1.76)	2.96 (1.49)	3.83 (1.74)
Number of underlying conditions, median (inter quartile range)	3 (2)	3 (2)	4 (2)	3(2)	4 (2)
Anxiety	850 (72%)	166 (71%)	92 (66%)	430 (73%)	162 (74%)
Obesity	654 (54%)	118 (50%)	82 (58%)	344 (57%)	110 (50%)
Depression	415 (38%)	67 (30%)	41 (33%)	214 (39%)	93 (46%)
Low level of physical activity	420 (35%)	81 (34%)	68 (48%)	193 (32%)	78 (35%)
Heart failure	237 (21%)	39 (17%)	29 (22%)	112 (20%)	57 (27%)
Asthma	239 (20%)	34 (14%)	9 (6%)	157 (26%)	39 (18%)
Obstructive lung disease (OLD)	215 (18%)	40 (17%)	48 (34%)	54 (9%)	73 (33%)
Anaemia	92 (8%)	25 (11%)	15 (11%)	41 (7%)	11 (5%)
Reduced diffusion capacity	204 (17%)	10 (4%)	18 (13%)	92 (15%)	84 (38%)
Cardiac valvular heart disease	139 (12%)	37 (16%)	23 (16%)	47 (8%)	32 (14%)
Pulmonary emphysema	131 (11%)	15 (6%)	44 (31%)	17 (3%)	55 (25%)
Restrictive spirometry pattern	64 (5%)	22 (10%)	12 (9%)	26 (4%)	4 (2%)
Atrial fibrillation or other arrhythmia	49 (4%)	18 (8%)	9 (6%)	13 (2%)	9 (4%)
Ischemic heart disease	18 (2%)	6 (3%)	6 (4%)	4 (1%)	2 (1%)
Pulmonary fibrosis (PF) or Fibrotic ILA (FILA)	14 (1%)	4 (2%)	1 (1%)	5 (1%)	4 (2%)
Underweight	9 (1%)	0 (0%)	2 (1%)	5 (1%)	2 (1%)

Values are presented as mean (standard deviation) or frequency (%). For definitions of underlying conditions and abbreviations see Table 1. Clinically significant breathlessness was defined as a modified Medical Research Council [mMRC] breathlessness scale rating ≥2, corresponding to having breathlessness when walking on level ground, or worse.

Compared with participants with <20 pack-years of smoking, participants with ≥ 20 pack-years of smoking had more airflow limitation (lower FEV_1_/FVC ratio), respiratory and cardiovascular diseases, and depression (Table 2).

### The AI developed diagnostic pathways

**Fig. 2:**
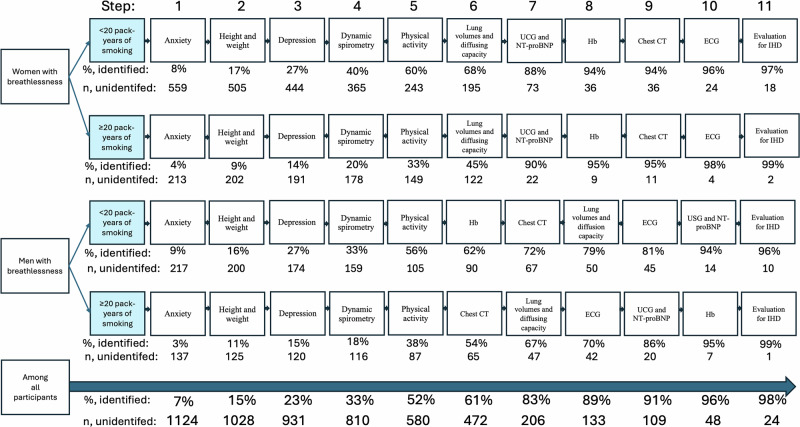
Diagnostic pathways with highest performance to identify underlying medical conditions in people with breathlessness with lowest cost. Diagnostic pathway to identify the underlying condition(s) to breathlessness with as low costs as possible developed using AI. Percentage corresponds to cumulative percentage of participants with all underlying conditions identified. Underlying conditions are presented in Table 1. Breathlessness was defined as modified medical research council breathlessness (mMRC) rating ≥ 2 (breathless when walking on level ground or worse). Evaluation for IHD includes electrocardiogram and computer tomography or stress echocardiography. Abbreviations: CT computer tomography, Hb haemoglobin, IHD ischemic heart disease, NT-pro-BP = N-terminal pro b-type natriuretic peptide; ECG echocardiography. UCG Echocardiogram.

The final diagnostic pathways stratified by sex and smoking history developed by the AI agent are shown in Fig. 2. For all participant groups, the initial evaluations (or order of effectiveness in relation to costs) were (% of participants where all their underlying conditions were identified): the level of perceived anxiety (7%), BMI (15%), depression (23%), dynamic spirometry (33%), and level of physical activity (52%). After those initial steps, the diagnostic pathway took different paths for the participant groups with more specialised evaluations including evaluations of lung volumes and diffusion capacity, chest CT, and assessment for anaemia (Hb) (Fig. 2). Chest CT was suggested earlier in the diagnostic pathway for men than for women. Conversely, dynamic spirometry and DLCO, ECG and NT-pro-BNP were suggested before chest CT among women (Fig. 2).

The cumulative percentage of participants that had all underlying factors identified after each step of the diagnostic pathways are shown in Fig. 2. After the initial five steps, 52% of the participants had all their underlying medical conditions identified, which increased to 61% after step six, 83% after step seven, 89% after step eight, 91% after step nine, 96% after step ten. At the end of the diagnostic pathway (step eleven), 98% of participants had all their underlying medical conditions in the analysis identified. Men and women with ≥20 pack-years of smoking had considerably lower % of all health conditions identified by the initial steps, compared to men and women with <20 pack-years of smoking, but at the end of the diagnostic pathway, the percentage of all health conditions were more similar between the groups. The last step, evaluation for ischemic heart disease (stress-Echo or CT coronaryangiography) only added two percentage points to the number of participants with all underlying conditions identified. The cost of the final diagnostic pathway (based on the mean cost per participant after no more underlying conditions could be identified) was 359 (standard deviation: 153) Euros (Supplementary materials).

### Consensus of clinical diagnostic pathway

**Fig. 3:**
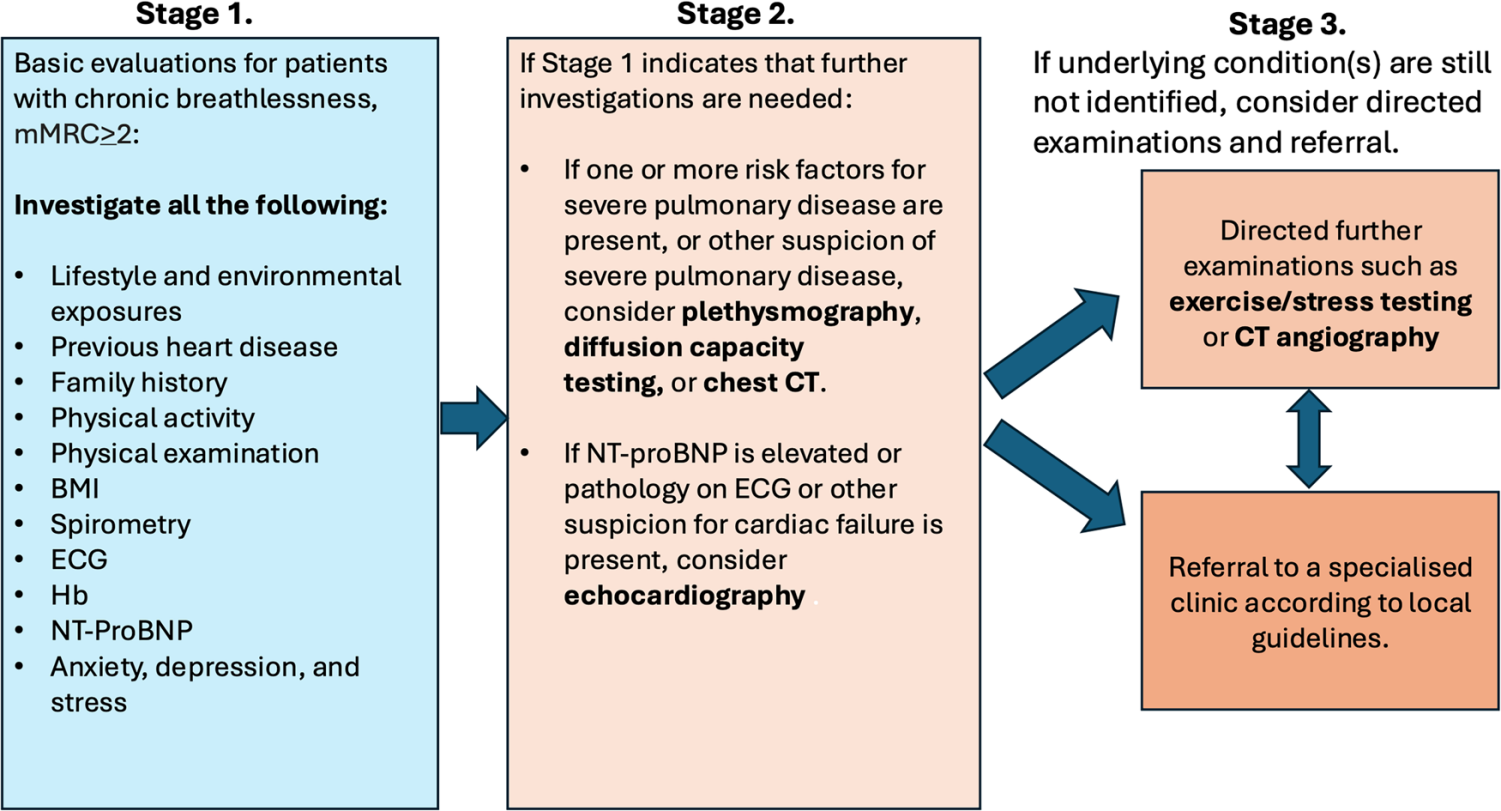
Proposed clinical diagnostic pathway to identify underlying medical conditions in people with breathlessness. The consensus for the suggested clinical diagnostic pathway for health care was made after a meeting with the authors. Physical examination encompasses the assessment of resting heart rate and rhythm, oxygen saturation, respiratory rate, and blood pressure. It also involves observing general appearance, including cyanosis, jaundice, and altered breathing patterns (increased use of accessory muscles). Peripheral edema should be evaluated, and lung and cardiac sounds should be auscultated. Evaluation of lifestyle factors include asking about smoking status and the use of vaporisers. Environmental exposures include asking the patients about cooking methods at home and occupational exposure to dust, smoke, chemicals, and mold.” Abbreviations: CT computer tomography; D_LCO_ diffusing capacity for carbon monoxide; Hb haemoglobin; NT-pro-BP = N-terminal pro b-type natriuretic peptide; ECG echocardiography; UCG Echocardiogram.

After a meeting with the clinical co-authors, a consensus for a suggested clinical diagnostic pathway for health care was made incorporating the new information from the AI generated algorithms (Fig. 3). At stage one, a throughout assessment of the following risk factors and biomarkers should be assessed to both diagnose and also improve pre-test probability for further testing: lifestyle and environmental exposures, past medical history, family history, physical activity, physical examination, BMI, followed by initial investigations of spirometry, ECG, Hb, and NT-pro-BNP, patient reported outcomes for anxiety and depression. For more details about the physical examination, and questions about lifestyle and environmental exposures, see captions for Fig. 3.

At stage two, the probability of additional conditions should be considered based on findings from step one and can include plethysmography, DLCO and Chest CT if suspicion of severe pulmonary disease, or UCG if suspicion of heart disease based on NT-proBNP or pathology on ECG. At stage three, directed examinations including exercise/stress test or CT angiography and referral to specialised clinic according to local guidelines are proposed. It is estimated that approximately 60% of the participants would have all their underlying medical conditions identified in stage one in the final diagnostic pathway, reaching approximately 83% if the NT-proBNP test included in stage one would be followed up by echocardiography in stage two to diagnose suspected heart failure. The cost of stage one of the final diagnostic pathway is estimated to be 143€ (not including the cost of the primary care visit) with an additional 193€ for diffusion capacity, 288€ for chest CT, and 340€ each for electrocardiography, exercise/stress testing or CT angiography.

## Discussion

### Main findings

This study presents the first evidence-based diagnostic pathways specifically designed to identify the widest range of underlying medical conditions associated with breathlessness in order of effectiveness and cost. Using a novel AI approach, we present diagnostic pathways optimised to identify potential underlying conditions in people with breathlessness, taking into consideration the prevalence of health conditions, the person’s sex and smoking history, as well as the economic costs. With a total of 16 potential underlying conditions, 98% of the participants had all underlying conditions identified by the pathway to a mean cost of approximately 359 Euro for the evaluation per participant (excluding the cost for primary care visit). From the AI-generated diagnostic pathway we interpreted the AI findings into a staged approach to diagnostic investigations for clinical care.

### What this study adds

The diagnostic pathways were created using AI methodology to specifically account for the prevalence of the underlying conditions in relevant pre-specified groups (men/women with none or low/more smoking exposure) using a random population-based sample of middle-aged people, and keeping costs as low as possible^19,27^. Using the AI generated (data driven) best pathways, a diagnostic pathway for use in clinical practice, focusing on primary care, was proposed by the multidisciplinary author group. The optimal sequences were overall similar between the groups, which informs on the importance of these tests for evaluating breathlessness in clinical practice. However, different algorithms are needed for men and women. In women, we found no evidence of added value of performing chest CT as an early step as the % of identified health conditions in relation to the cost of chest CT was considered not valuable by the AI agent.

When comparing our diagnostic pathway with other existing ones, the key benefit is that ours is based on empirical evidence from population data and health care costs. Notably, when comparing with the multifactorial NHS-E pathway^20^, the first stage is similar to ours, but our diagnostic pathway recommends all tests being done at the first stage, whereas the NHS-E recommends tests in the first stage according to clinical judgement. Based on the % of identified health conditions in our diagnostic pathway at the first stage, we believe that there is a diagnostic and economic motivation for a thorough assessment of breathlessness that includes evaluation for the most common underlying conditions of the symptom early on. Compared to other diagnostic pathway studies of breathlessness, our work contributes to the knowledge of the order of tests, diagnostic utility, and cost, which can be especially valuable when adjusting the guidelines for different health care settings. Additionally, our diagnostic pathway prioritises spirometry – an important message to the primary care. Our strategy included a CT thorax for men after conducting tests that can be done in primary care setting. This is different from an earlier strategy^28^ which suggests CT thorax only as a last step. However, we still believe that there is a value in performing CT thorax early on among both men and women as we consider that the conditions that can be identified through CT scans are very dangerous in terms of mortality and morbidity^29,30^, where earlier diagnostics and treatment are of importance.

The findings highlight the importance of early spirometry in the clinical evaluation of breathlessness, and that clinical evaluation of the symptom should include tests to identify respiratory diseases early on^15^. Over half of middle-aged adults can have all their diagnoses for chronic breathlessness identified with a simple but thorough suite of investigations in primary care - spirometry is a key investigation out performing any other by effectiveness and cost and nearly two in five patients with chronic breathlessness will have a lung disease easily identified in primary care. This is important knowledge, since access to high quality spirometry is often lacking - especially in primary care where it is often not even used or considered^17^. We also provide evidence supporting respiratory diseases as important to overall evaluate earlier than heart diseases such as heart failure or IHD for patients suffering from breathlessness. Obesity, anxiety, and depression and physical inactivity were included as potential underlying conditions for breathlessness^8,31^ in our study, but are not always included in earlier diagnostic pathways^15,16^. The latter is in spite of these conditions being modifiable with treatments.

The percentage of identified associated conditions among the participants were high after tests that can be done in primary care, meaning that in the majority cases (52%), only a primary care visit is needed to identify all underlying medical conditions in relation to breathlessness for a patient. The pathway can be used for patients in primary care with similar backgrounds as the participants in our study as the pathway is based on evidence of underlying conditions among the general population.

### Strengths and limitations

Strengths of this study include the large population-based sample of people with clinically relevant breathlessness (mMRC ≥ 2) that is considered representative of the Swedish middle-aged general population^32^. Importantly, this is a unique cohort whereby all participants in our study went through all tests and in large numbers, in contrast to observational studies in people seeking care or specific clinical groups, which lowers the risk of bias from health care-seeking behaviours, confounding by indication by the indications and implicit and confirmation bias from clinicians.

Limitations include that the study assumes that all associated conditions contribute equally to a person’s breathlessness, and the tests in the AI developed pathway did not prioritise tests based on how dangerous the underlying conditions are. However, a diagnostic pathway that prioritise tests only based on how dangerous potential underlying conditions are, would most likely results in a diagnostic pathway with a high ratio of false positives resulting in unnecessary worriedness among patients and increased health care costs. In clinical practice, red flags should be prioritised as usual. The percentage of missing data on health condition was small, but the participants who had missing value were assumed to not have the underlying condition which might overestimate the percentage of participants that had all conditions identified. Because of the fact that there were only a few participants left at the last steps of the AI-developed pathway, the generalisability of the tests in the last steps of the AI-developed pathway should be lower than in the earlier steps as only a few participants are evaluated at those steps. The lack of existing reports of the costs of evaluating breathlessness in primary care make it challenging to compare our estimated cost of evaluating the symptom with existing strategies.

The findings are based on the availability and cost of tests and underlying conditions included in our study, but the findings have plausible external validity for high-income settings. Tests such as Fractional Exhaled Nitric Oxide (FeNo), blood eosinophils, and Cardiopulmonary Exercise Test (CPET) were not included but could add diagnostic value. The findings also pertain to middle-aged people, and additional diagnostic pathways should be developed for younger and older age groups, respectively. This is especially important since the underlying conditions contributing to breathlessness most likely vary between age groups, and future studies should use larger datasets with broader age groups. Future evaluation strategies of breathlessness can then benefit from using age groups as arms in a diagnostic pathway together with sex and smoking history.

### Implications

We propose an evidence-based diagnostic pathway to inform clinical evaluation of people with chronic breathlessness, based on the prevalence of underlying conditions and evaluation costs. Our overall suggestion is that all patients suffering from breathlessness should be offered a primary care visit including a physical examination, followed by an objective examination of their pulmonary function including spirometry, then cardiac examinations, followed by blood samples in contrast to common practice. We suggest that these basic steps, which identify a large proportion of common underlying conditions, are performed before patients are referred for specialised investigations or to specialised clinics. Clinicians should be aware of the potential sex differences of priority investigations after the initial steps. We acknowledge that there are discrepancies in the routine availability, and costs of specific tests which are context and country-specific, and we suggest adjusting the pathway to meet the needs accordingly. If some tests unavailable, the diagnostic pathway can instead inform the clinician which test is most probable to identify a condition in the specific patient groups in the specific setting. Combined with clinical evaluation, pre-test probability for certain medical conditions should also direct the evaluation. As multiple underlying conditions are often present, shown in the present analysis, the proposed diagnostic pathway could save time and money for patients and the health care system, and improve the evaluation and management of people with breathlessness.

Important research steps include studies validating the pathway in other settings and study populations. Future studies can develop diagnostic pathways with other goals such as prioritising high risk underlying conditions, prioritising underlying conditions with the highest treatment potential or the highest healthcare burden whilst undiagnosed, or prioritising underlying conditions that decrease quality of life. As this is the first study to develop a diagnostic pathway for any health condition or symptom using AI, our study can inspire using a similar approach for developing diagnostic pathways for other symptoms or health conditions.

## Conclusion

We present an evidence-based clinical evaluation pathway developed using AI methodology to identify as many underlying conditions effectively and at lowest cost for healthcare systems. The diagnostic pathway informs optimized cost-effective clinical evaluation of people with chronic breathlessness.

## Supplementary information

Supplmentary materials

## Data Availability

The SCAPIS dataset can be accessed by interested researchers. All studies need ethical permission from the Swedish Ethical Review Authority and the SCAPIS board. See http://www.scapis.org/ for more information about access to the SCAPIS dataset. The code used in this study can be given to collaborators upon reasonable request.
