# Supplementary material for: Using AI to determine optimal cost-effective diagnostic pathways for chronic breathlessness
Source: NPJ Prim Care Respir Med. 2026 Aug 13;36:57. doi: 10.1038/s41533-026-00548-9 (PMC13473488; doi:10.1038/s41533-026-00548-9)
Supplement: Supplementary file 1 [file 41533_2026_548_MOESM1_ESM.docx]

**SUPPLEMENTARY MATERIALS**

**Supplementary Figure S1 – Participant flow chart**

 Exclusion criteria for the present analysis were missing data on breathlessness (mMRC), or inability to walk for reasons other than breathlessness.

**Dynamic spirometry**

The dynamic spirometry was performed using a Jaeger MasterScreen PFT (Carefusion, Hoechberg. Germany) 15 min after inhaling 400 μg salbutamol in accordance with the standards of the American Thoracic Society (ATS) and the European Respiratory Society (ERS).

**Handling of missing values**

Participants who had missing values (mean: 3%, range: 0 – 9%) on health conditions were assumed to not have that condition.

**Reinforcement learning agent**

An RL agent (or “model”) is a one instance of the algorithm that learns a task and we used the novel RL algorithm Advantage Actor critic (A2C) which has previously been implemented for multiple advanced tasks. The simulated environment gives the agent information about the task, in our case, which patient group a patient belongs to, and which tests have already been performed. The environment also gives information about what happens after a test has been performed, such as if the agent has identified all underlying conditions and how well the agent performed. A reward score, as defined below, is given to the agent of how well it performed and through trial and error the agent uses this to improve itself. The reward score was defined as the summarised costs of the tests that were not used after all underlying conditions had been identified for the participant divided by the total cost of all available tests.

**Evaluation of the reinforcement agent.**

To evaluate the generalisability of the agent, the data was divided into sets for training (80%), testing (10%), and validation (10%). We tuned the hyperparameters (settings of the agent that can be tuned to reach better performance) by training the agent 100 times using random grid search on the training set and evaluated on the test set. The hyperparameters evaluated were learning rate (range: 0.00001 to 0.001) and gamma (range: 0.9000 to 0.9999). The final learning rate selected was 0.00056, and final gamma selected was 0.9394.

The final agent was evaluated on the validation set in terms of the mean cost per participant for all tests that were needed to identify all underlying health conditions in the dataset. The validation set was kept separated from the agent during the whole training phase. By this approach, we can measure the agent´s ability to generalise to data it has not seen before, and we can therefore better generalise for breathless patients visiting health care. Two other comparable algorithms were also compared: Deep Q-learning (DQN) and Proximal Policy Optimization (PPO) which were tested with the same procedure. The DQN and PPO performed worse than the A2C algorithm on the test set in terms of mean cost per participant.

The agent was also evaluated by conducting the same process five times with re-randomisation of the data splits with new versions of the agent trained from scratch each time, which resulted in similar findings in each re-randomisation.

As a baseline for measurement of performance, we created a diagnostic pathway based on tests chosen from lowest to highest costs which resulted in a modest lower mean cost per participant (train €305: test: €286, validation: €301 euro), but with a considerably lower diagnostic performance compared to our diagnostic pathway: only 62% of all participants had all their underlying conditions identified after step 7 in comparison to 83% in our diagnostic pathway when evaluated on the whole dataset. This was similar across all patient groups.

The final trained agent performed (based on the mean cost per participant after no more underlying conditions could be identified) was similar in the training (mean: €302; SD: €116), test (mean: €365; SD: €152) and validation set (mean: €359; SD: €153). Taken together, the evaluations supported that the agent is robust and can generalise for unseen participants**.**

To be able to present the developed pathways for the participant groups, we instructed the agent to perform tests on one participant from each participant group who had none of the underlying conditions defined in our study. By this method, we present in which order the agent would use the tests if it would need to use all tests available in the study. All analyses were conducted in Python (version 3.9.15) using the libraries Gymnasium and Stable baselines 3.

**Supplementary Table S2. Characteristics of participants in Train, Test, and validation set.**

|  | **Train** | **Test** | **Validation** |
| --- | --- | --- | --- |
| **n** | 967 | 121 | 121 |
| **Age** | 58.09 (4.40) | 58.52 (4.20) | 58.52 (4.52) |
| **BMI (kg/m^2^)** | 30.92 (6.04) | 31.38 (5.80) | 31.27 (5.47) |
| Pack years of smoking | 13.69 (16.98) | 13.72 (17.92) | 14.79 (19.01) |
| **FVC (L)** | 3.57 (0.86) | 3.50 (0.84) | 3.47 (0.82) |
| **FVC (%pred)** | 102.02 (14.52) | 102.78 (15.36) | 100.07 (16.61) |
| **FEV_1_ (L)** | 2.70 (0.70) | 2.63 (0.67) | 2.62 (0.68) |
| **FEV_1_ (%pred)** | 97.45 (17.79) | 97.20 (17.28) | 95.83 (20.06) |
| **FEV_1_/FVC ratio** | 0.76 (0.09) | 0.75 (0.09) | 0.76 (0.09) |
| **Underlying medical conditions** |  |  |  |
| Number of underlying conditions, mean (standard deviation) | 3.24 (1.64) | 3.41 (1.78) | 3.28 (1.62) |
| Number of underlying conditions, median (inter quartile range) | 3 (2) | 3 (2) | 3 (2) |
| **Education level** |  |  |  |
| No elementary school | 20 (2.1) | 2 (1.7) | 2 (1.7) |
| Elementary school | 164 (17.1) | 21 (17.5) | 25 (20.7) |
| Upper secondary school or professional education | 504 (52.5) | 58 (48.3) | 56 (46.3) |
| University degree | 272 (28.3) | 39 (32.5) | 38 (31.4) |
| **Current smoking status** |  |  |  |
| Regular | 135 (14.2) | 15 (12.6) | 19 (15.7) |
| Occasional | 42 (4.4) | 8 (6.7) | 5 (4.1) |
| Former | 407 (42.8) | 48 (40.3) | 46 (38.0) |
| Never | 368 (38.7) | 48 (40.3) | 51 (42.1) |
| **Underlying medical conditions** |  |  |  |
| Obstructive lung disease (OLD) | 170 (18.1) | 27 (22.7) | 18 (15.3) |
| Restrictive spirometry pattern | 49 (5.2) | 5 (4.2) | 10 (8.4) |
| Asthma | 194 (20.6) | 22 (18.6) | 23 (19.2) |
| Anaemia | 71 (7.4) | 14 (11.6) | 7 (5.8) |
| Obesity | 514 (53.2) | 68 (56.2) | 72 (59.5) |
| Underweight | 7 (0.7) | 1 (0.8) | 1 (0.8) |
| Atrial fibrillation or other arrhythmia | 38 (4.0) | 8 (6.8) | 3 (2.5) |
| Low level of physical activity | 340 (35.6) | 40 (33.9) | 40 (33.3) |
| Heart failure | 191 (21.0) | 23 (20.0) | 23 (20.0) |
| Pulmonary fibrosis (PF) or Fibrotic ILA (FILA) | 12 (1.3) | 2 (1.7) | 0 (0.0) |
| Depression | 338 (38.2) | 38 (35.2) | 39 (37.1) |
| Anxiety | 671 (70.9) | 88 (75.9) | 91 (77.1) |
| Pulmonary emphysema | 95 (10.2) | 20 (16.8) | 16 (13.6) |
| Ischemic heart disease | 15 (1.6) | 1 (0.8) | 2 (1.7) |
| Reduced diffusion capacity | 157 (17.5) | 24 (20.9) | 23 (19.5) |
| Cardiac valvular heart disease | 113 (12.5) | 14 (12.2) | 12 (10.4) |
